# Establishment of a cone photoreceptor transplantation platform based on a novel cone-GFP reporter mouse line

**DOI:** 10.1038/srep22867

**Published:** 2016-03-11

**Authors:** Sheila Smiley, Philip E. Nickerson, Lacrimioara Comanita, Narsis Daftarian, Ahmed El-Sehemy, En Leh Samuel Tsai, Stuart Matan-Lithwick, Keqin Yan, Sherry Thurig, Yacine Touahri, Rajiv Dixit, Tooka Aavani, Yves De Repentingy, Adam Baker, Catherine Tsilfidis, Jeff Biernaskie, Yves Sauvé, Carol Schuurmans, Rashmi Kothary, Alan J. Mears, Valerie A. Wallace

**Affiliations:** 1 Ottawa Hospital Research Institute, 501 Smyth Road, Ottawa, Ontario, K1H 8L6, Canada; 2 Department of Ophthalmology, University of Ottawa, Ottawa, ON K1H 8M5, Canada; 3 Departments of Biochemistry, Microbiology and Immunology, University of Ottawa, Ottawa, ON K1H 8M5, Canada; 4 Department of Cellular and Molecular Medicine, University of Ottawa, Ottawa, ON K1H 8M5, Canada; 5 Department of Medicine, University of Ottawa, Ottawa, ON, K1H 8M5, Canada; 6 Division of Vision Science, Department of Ophthalmology and Vision Science, Krembil Research Institute, University Health Network, Toronto, ON M5T 2S8, Canada; 7 Department of Laboratory Medicine and Pathobiology, University of Toronto, Toronto, ON M5S 2J4, Canada; 8 Department of Biochemistry and Molecular Biology, Hotchkiss Brain Institute and Alberta Children’s Hospital Research Institute, University of Calgary, 3330 Hospital Drive NW, Calgary, T2N 4N1, Canada; 9 Department of Comparative Biology and Experimental Medicine, Hotchkiss Brain Institute and Alberta Children’s Hospital Research Institute, University of Calgary, 3330 Hospital Drive NW, Calgary, T2N 4N1, Canada; 10 Department of Ophthalmology and Visual Sciences, University of Alberta, Edmonton, AB T6G 2H7, Canada; 11 Ocular Tissue Engineering Research Center, Shahid Beheshti University of Medical Sciences (SBMU), Tehran, Iran

## Abstract

We report successful retinal cone enrichment and transplantation using a novel cone-GFP reporter mouse line. Using the putative cone photoreceptor-enriched transcript *Coiled-Coil Domain Containing 136* (*Ccdc136*) GFP-trapped allele, we monitored developmental reporter expression, facilitated the enrichment of cones, and evaluated transplanted GFP-labeled cones in wildtype and retinal degeneration mutant retinas. GFP reporter and endogenous *Ccdc136* transcripts exhibit overlapping temporal and spatial expression patterns, both initiated in cone precursors of the embryonic retina and persisting to the adult stage in S and S/M opsin^+^ cones as well as rod bipolar cells. The trapped allele does not affect cone function or survival in the adult mutant retina. When comparing the integration of GFP^+^ embryonic cones and postnatal *Nrl*^−/−^ ‘cods’ into retinas of adult wildtype and blind mice, both cell types integrated and exhibited a degree of morphological maturation that was dependent on donor age. These results demonstrate the amenability of the adult retina to cone transplantation using a novel transgenic resource that can advance therapeutic cone transplantation in models of age-related macular degeneration.

Cone photoreceptors are the light sensitive cells in the retina that mediate colour and high acuity vision. Because these cells are enriched in the macula of the human retina, their degeneration results in a significant impairment of the central visual field. Cone degeneration can occur as a consequence of mutations in cone-specific genes or can be secondary to rod degeneration, with the most prevalent cone degenerative condition being age-related macular degeneration (AMD). AMD is thought to involve an inflammatory component coupled with dysfunction of the choroid and retinal pigment epithelium (RPE), which is essential for structural, trophic and metabolic support of photoreceptors (reviewed in^1^). At present, the only widely available treatment for AMD targets neovascularization that develops in a small subset of AMD patients. Newer approaches to treatment, including RPE transplantation, are at pre-clinical and early stage clinical trials^2^; unfortunately, this approach cannot reverse vision loss after cone degeneration, necessitating the development of novel strategies, such as cone transplantation to restore vision to the AMD retina.

The feasibility of photoreceptor transplantation to repair and restore retinal functions in mice has been demonstrated with rod engraftment^3^,^4^,^5^,^6^. A key advance in this field was the demonstration that immature postmitotic rod precursors, as opposed to dividing retinal progenitor cells or mature rods, exhibit the best integration potential in the adult retina^4^. In contrast to this progress on rod transplantation, investigation of cone transplantation has been remarkably more challenging, primarily due to the paucity of transgenic reporter lines that mark the cone lineage. Several GFP-reporter mouse strains specific to photoreceptors exist, but they are either rod-specific (*Nrl-EGFP*^7^), mark rods and cones (*Crx-GFP*^8^) which does not permit direct assessment of cone transplantation^9^, or are expressed at low levels in cone precursors and induce cone degeneration (OPN1LW-GFP)^10^,^11^.

In the mouse retina, cones represent 3% of all photoreceptors, of which 7% are S and 93% M types, responding to short wave (blue) and medium wavelength (green) light, respectively. Cones are generated between embryonic day 11 (E11) and E18 in the mouse, and like rods, are produced as postmitotic precursors (reviewed in^12^) that are marked by expression of pan-photoreceptor transcription factors Otx2 and Crx, as well as RXRγ and TRβ2, which regulate cone opsin expression^13^,^14^. The induction of late stage differentiation markers, including proteins involved in the phototransduction cascade, begins postnatally^12^. The maturation of both cone subtypes is characterized by the development of photosensitive outer segments (OS), synapse formation with horizontal and bipolar cells at the outer plexiform layer, and assembly of the phototransduction apparatus.

We describe the development of a novel GFP-reporter mouse line with a gene trap insertion into the *Coiled-coil domain containing 136* (*Ccdc136*)/*nasopharyngeal carcinoma-associated gene 6* (*NAG6*) locus. Ccdc136 is predicted to be a single-pass membrane protein with coiled-coil domains. Although the function of Ccdc136 is largely unknown, it is frequently deleted in nasopharyngeal, gastric, breast and ovarian cancers, suggesting a role in tumor suppression^15^. *Ccdc136* expression has been reported in the outer nuclear layer (ONL) of cone only *Nrl*^−/−^ and cone/rod hybrid *Nr2e3*^−/−^ mouse retinas^16^,^17^,^18^. Here we report that in the *Ccdc136*^*GFP*^ mouse, reporter gene expression from the *Ccdc136*^*GFP*^ allele is initiated in embryonic cone precursors located at the apical surface of the retina and marks S and S/M cones, as well as rod bipolar cells in the adult. Consistent with expression in cones in the adult retina, the GFP reporter was expressed in the majority of cells in the photoreceptor layer in the cone-dominant *Nrl*^−/−^ background^19^. Transplanted GFP tagged cones from both embryonic wildtype and postnatal *Nrl*^−/−^ mice integrated into the photoreceptor layer of adult recipient mice and adopted photoreceptor morphologies. This study describes a reporter strain that can be used to identify cone precursors that are competent to integrate into recipient retinas, demonstrating a powerful resource for monitoring early cone development and as a tracer for cone transplantation.

## Results

### Gene trap of the *Ccdc136* locus

The *Ccdc136* gene is located 3′ of *Opn1sw* (S opsin) on mouse chromosome 6, consists of 15 exons spanning 31.1 kb (Fig. S1A) and encodes a 134 kDa protein that is highly conserved between human and mouse. To understand the significance of *Ccdc136* expression in the retina, and specifically in cone photoreceptors, we took advantage of a gene trap ES cell line generated by the insertion of a pUPA GFP reporter vector 3′ of exon 2 in the *Ccdc136* gene (obtained from the CMHD, Toronto) (Fig. S1B). *Ccdc136*^*GFP/GFP*^ mice derived from this ES line were viable and fertile and the position of the gene trap insertion at the *Ccdc136* locus was confirmed by PCR analysis of genomic DNA (see Methods). RT-Q-PCR with primers spanning exons 2 and 3 of *Ccdc136* amplified a product in the *Ccdc136*^*GFP/GFP*^ retina (albeit significantly reduced relative to wildtype littermates), suggesting the possibility that the trapped allele could produce wildtype transcripts where the gene trap sequences are spliced out (Fig. S1C). The presence of putative wildtype transcript in the *Ccdc136*^*GFP/GFP*^ retina was further confirmed by amplification of products with primers specific for the 3′ end of the transcript (Fig. S1C). We were unable to assess Ccdc136 protein levels using commercially available antibodies, however, the detection of low levels of potentially functional *Ccdc136* transcripts in *Ccdc136*^*GFP/GFP*^ mice raised the possibility that this strain is not a null allele for *Ccdc136*.

**Figure 1:**
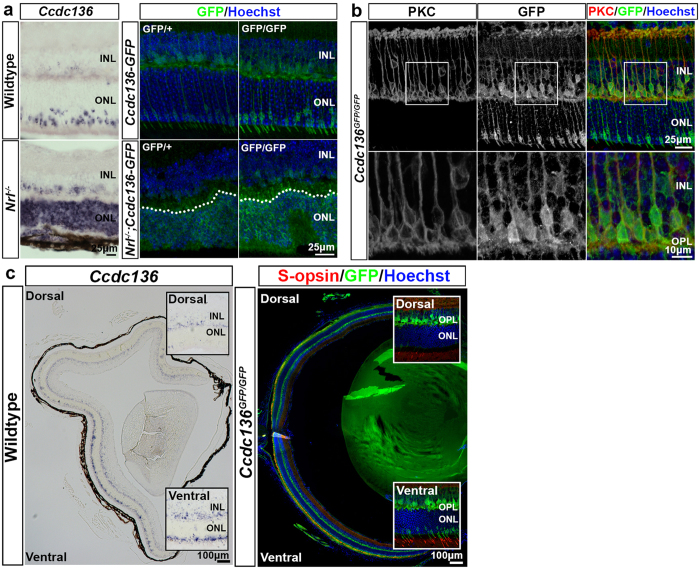
*Ccdc136* expression in the adult retina. (**a**) Localization of *Ccdc136 *mRNA and GFP reporter expression in adult retina from wildtype (left column), *Ccdc136*^*GFP*/+^ and *Ccdc136*^*GFP/GFP*^ mice. Images are from the central retina at the level of the optic nerve head. The dashed line indicates the border of the ONL in *Nrl*^−/−^ retinas. (**b**) Co-localization of the immunofluorescence for anti-PKC and anti-GFP in the INL of adult retina of *Ccdc136*^*GFP/GFP*^ mice with higher magnification images (bottom row). (**c**) Ventral-to-dorsal gradients of *Ccdc136* transcript in wildtype and S-opsin and the GFP reporter in *Ccdc136*^*GFP/GFP*^ adult retinas with higher magnification images in insets. INL, inner nuclear layer; IS/OS, inner segments/ outer segments; ONL, outer nuclear layer; OPL, outer plexiform layer.

**Figure 2:**
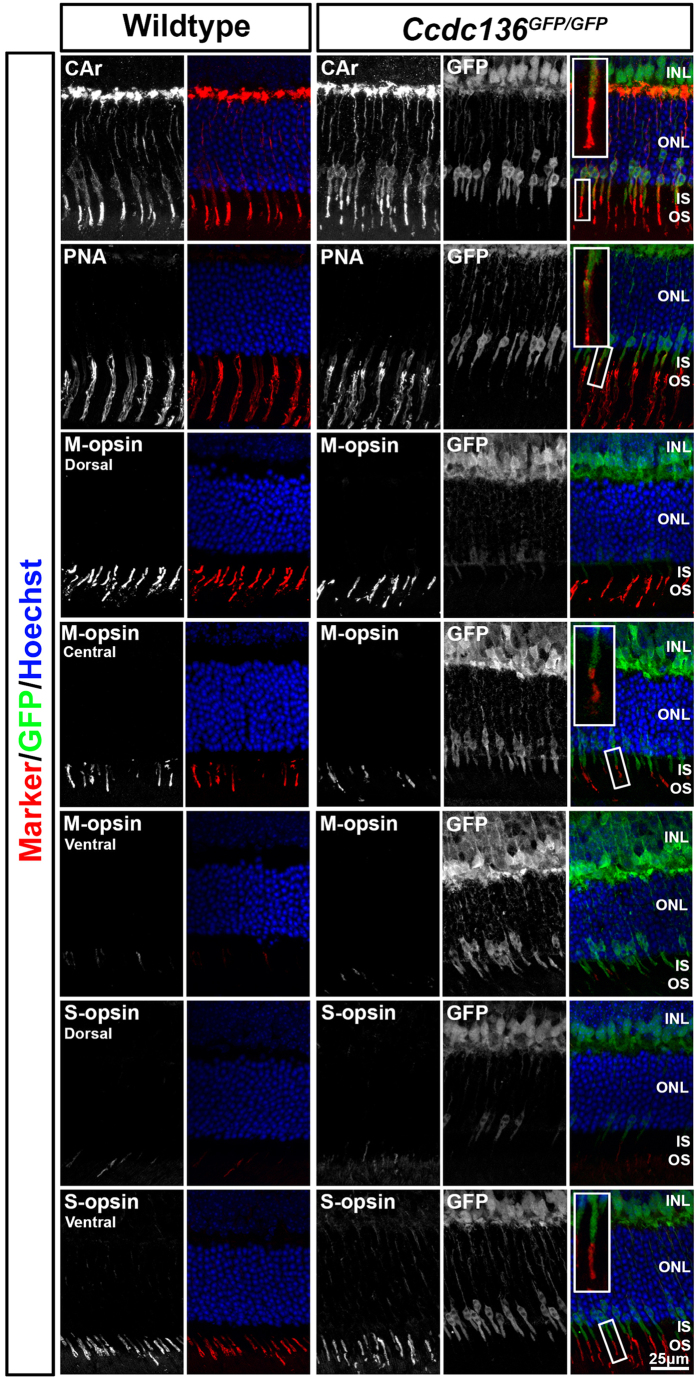
Expression of cone photoreceptor markers in adult wildtype and *Ccdc136*^*GFP/GFP*^ retinas. Cone arrestin is a pan cone marker. PNA labels cone inner and outer segments. M opsin labels outer segments of M cones, expressed more in the dorsal than ventral retina. S opsin labels outer segments of S cones, expressed more in the ventral than dorsal retina. Insets show outer segments of GFP^+^ cones in *Ccdc136*^*GFP/GFP*^ retina co-expressing cone markers. INL, inner nuclear layer; IS, inner segments; ONL, outer nuclear layer; OS, outer segments.

In the adult retina the intensity of GFP staining correlated with *Ccdc136*^*GFP*^ dosage (Fig. 1a). The pattern of GFP reporter expression and *Ccdc136* transcripts overlapped, with both signals localizing to subsets of cells in the ONL and inner nuclear layer (INL) (Fig. 1a) and both being increased in the ONL in the *Nrl*^−/−^ background (Fig. 1a), which is consistent with the reported enrichment of *Ccdc136* transcripts in this mutant^16^,^17^,^18^. GFP^+^ cells in the INL were rod bipolar cells, based on co-staining with PKC (Fig. 1b). GFP^+^ cells in the ONL exhibited the same dorsal to ventral distribution as S opsin (Fig. 1c), which is consistent with the pattern of endogenous *Ccdc136* expression and suggests that these genes are co-regulated^18^ (Fig. 1c). GFP^+^ cells in the ONL have the characteristic morphology of cone photoreceptors, with GFP labeling in apical processes extending as far as the inner segment (IS)/outer segment (OS) boundary and basal processes terminating at the outer plexiform layer (Fig. 1a). All of the GFP^+^ cells in the ONL co-stained with pan-cone markers, including cone arrestin and Peanut Agglutinin (PNA) (Fig. 2). While GFP expression was low to undetectable in M opsin^+^ cells in the dorsal retina, it did overlap with M opsin-expressing cells in the central retina and all S opsin^+^ cells, identifying the GFP cones as belonging to the S and double S/M cone subsets (Fig. 2). Finally, there was no overlap between GFP and rhodopsin staining in single cell dissociates from adult *Ccdc136*^*GFP*^ mice (Fig. S2A), further confirming the restriction of the reporter to cones in the ONL. These data indicate that *Ccdc136* expression marks S and S/M cones in the ONL and rod bipolar cells in the INL and that the GFP reporter from the trapped allele is a faithful surrogate for endogenous *Ccdc136* gene expression.

Because cone degeneration has been observed in a previously characterized transgenic cone reporter line^11^, we examined cone number and function in *Ccdc136*^*GFP/GFP*^ mice. We found no evidence of cone degeneration in *Ccdc136*^*GFP/GFP*^ mice at 3 and 8 months (Fig. S3) or alterations of other retinal cell types based on the expression pattern of lineage specific markers (Fig. S2B). Moreover, *Ccdc136*^*GFP/GFP*^ mice exhibited normal photopic electroretinograms with no significant difference in response compared to wildtype mice (Fig. S4B–D). Taken together, the *Ccdc136*^*GFP*^ allele is compatible with cone viability and function.

### Developmental expression of *Ccdc136* reporter in relation to cone differentiation

**Figure 3:**
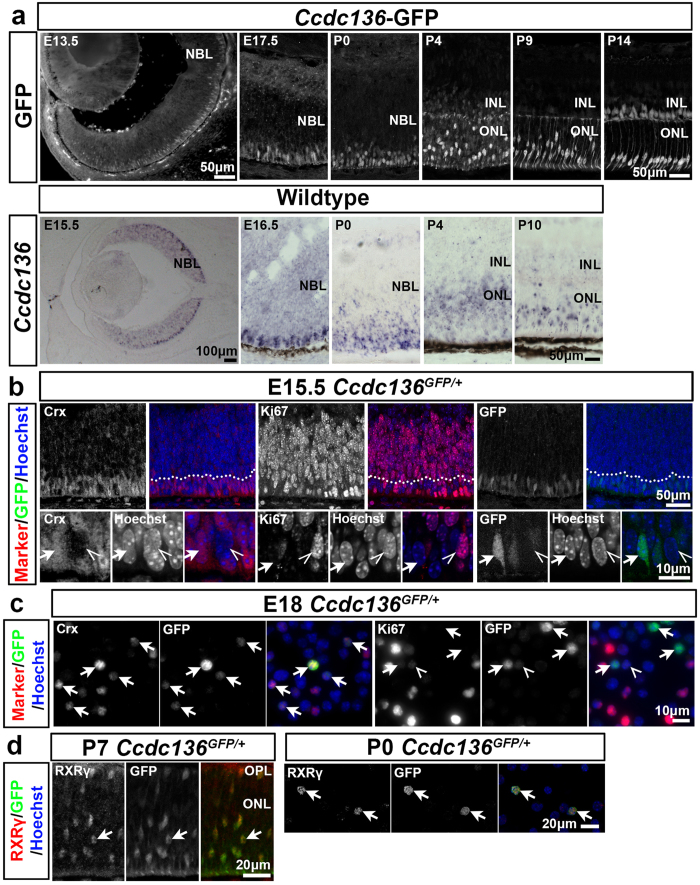
Expression of *Ccdc136* reporter in relation to cone differentiation. (**a**) GFP reporter expression in *Ccdc136*-GFP mice compared to *Ccdc136* mRNA expression in wildtype mice from embryonic day 13.5 (E13.5) to postnatal day 14 (P14). (**b**) Immunohistochemistry for a photoreceptor marker (Crx), proliferation marker (Ki67) and the GFP reporter in E15.5 *Ccdc136*^*GFP*/+^ retinas. The dashed line indicates the bottom 3 rows of nuclei with higher magnification images in the bottom row. Post-mitotic cells with condensed heterochromatin indicated by arrows. Mitotic cells with diffuse heterochromatin indicated by open arrowheads. (**c**) Expression of cell markers in E18.5 *Ccdc136*^*GFP*/+^ dissociated cells. GFP^+^ cells indicated by arrows. Rare cell expressing low levels of Ki67 and GFP indicated by open arrowhead. (**d**) Co-localization of *Ccdc136*-GFP reporter with the cone marker RXRγ in P7 *Ccdc136*^*GFP*/+^ retina (left) and in P0 *Ccdc136*^*GFP*/+^ dissociates (right). Arrows indicate double-labeled RXRγ^+^ GFP^+^ cells. INL, inner nuclear layer; NBL, neuroblast layer; ONL, outer nuclear layer; OPL, outer plexiform layer.

We next investigated when the *Ccdc136*^*GFP*^ reporter was activated in developing cones. At E13.5, GFP expression was induced in a central to peripheral gradient and localized to cells at the apical side of the retina, similar to the pattern of *Ccdc136* transcript (Fig. 3a), which is consistent with the onset of cone development. By E15.5 the majority of GFP^+^ cells were located within approximately 3 cell diameters from the apical surface and exhibited a teardrop shaped morphology with an apically oriented process (Fig. 3b, arrows). Embryonic rods and cones in other GFP reporter mice adopt a similar morphology^9^,^10^, which suggests that there is already some degree of maturation at the earliest stages of photoreceptor development. The apical region of the retina at this stage consists largely of postmitotic photoreceptor precursor cells, marked by Crx, and mitotic progenitors, marked by high levels of Ki67. Although technical restrictions prevented us from co-staining GFP and Crx in sections, the GFP^+^ cells are likely cone precursors and not cycling progenitors based on the following criteria: GFP^+^ and Crx^+^ cells have a similar pattern of condensed heterochromatin (Fig. 3b, arrows), which is distinct from the diffuse chromatin in mitotic Ki67^+^ cells (Fig. 3b, open arrowheads). The majority of GFP^+^ cells in single cell dissociates from E18-P0 retina co-stained with Crx and RXRγ, which marks embryonic and adult cones (Fig. 3c,d). While we detected rare cells expressing low levels of GFP and Ki67 in dissociated retinal cells (Fig. 3c, open arrowhead), these could represent cone precursors that are about to or that have just exited the cell cycle^20^. Based on cell and nuclear morphology, co-expression with cone and photoreceptor markers and the timing of GFP expression we conclude that the majority of GFP^+^ cells in the embryonic *Ccdc136*^*GFP/GFP*^ retina are cone precursor cells.

Another advantage of the *Ccdc136* reporter was that it allowed us to monitor cone photoreceptor maturation in the postnatal retina of *Ccdc136*^*GFP*^mice. At postnatal day 0 (P0), cells expressing *Ccdc136* transcripts and GFP were located on the apical side of the retina, however, after P0 we noted a change in the distribution of GFP^+^ cells and *Ccdc136* transcripts in the ONL (Fig. 3a). First, cone cell bodies, marked by intense GFP expression (Fig. 3a), changed from apical to more basal locations, consistent with the basal migration of cone nuclei that has been reported previously^21^. This basal movement of cone nuclei coincided with the presence of cone axon terminals at the border of the outer plexiform layer (Fig. 3a) and the cessation of proliferation in the central retina (Fig. S5). This nuclear displacement was also apparent at P7, where we observed RxRγ^+^GFP^+^ double positive cells located in more basal positions within the ONL (Fig. 3d). By P14, cone cell bodies re-established their adult-like apical distribution (Fig. 3a). The perinatal change in GFP^+^ nuclear positioning was mirrored by a similar change in the distribution of *Ccdc136* transcripts, however, without the same cellular resolution observed with the GFP reporter (Fig. 3a). These data show that this reporter line can track cone nuclear displacement at the single cell level and that this behaviour is coupled spatially and temporally with the onset of differentiation and synaptogenesis.

**Figure 4:**
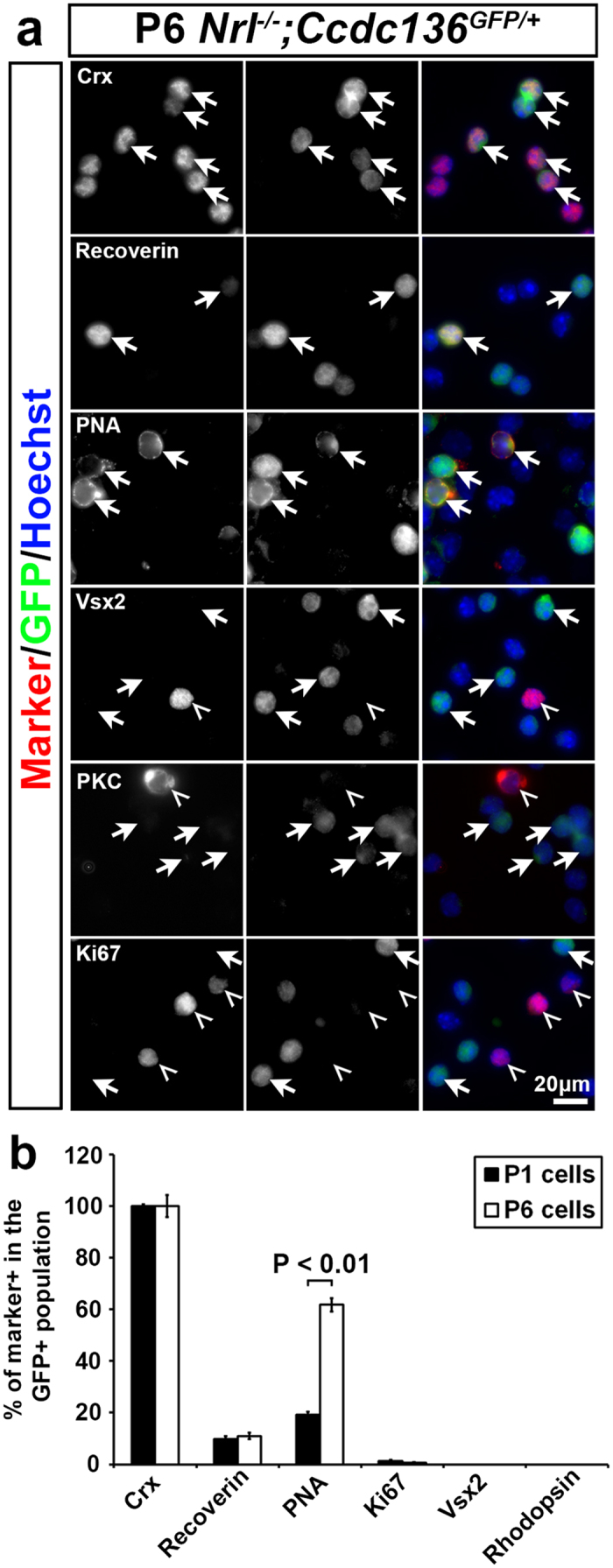
Characterization of postnatal cod-GFP cells. (**a**) Expression of cell markers in P6 *Nrl*^−/−^*;Ccdc136*^*GFP*/+^ dissociated cells. Crx and recoverin are photoreceptor markers, PNA is a cone specific marker, Vsx2 is a bipolar cell marker and Ki67 is a proliferation marker. (**b**) Percentage of cells showing positive immunostaining for indicated cell markers in the GFP^+^ cell population of P1 and P6 *Nrl*^−/−^*;Ccdc136*^*GFP*/+^ retinas. Cells were dissociated by papain treatment and immunostained for the marker and GFP. Five fields of view were taken for each condition containing at least 200 cells/view. Cells were counted and expressed as a proportion of the GFP^+^ cells. Bars represent mean + SEM; statistical significance assessed by paired t-test (n = 3).

As another approach to investigate the relationship between *Ccdc136*^*GFP*^ reporter expression and cone differentiation, we analyzed retinas of compound *Nrl*^−/−^;*Ccdc136*^*GFP*/+^ mice. Cone development and function in this line is indistinguishable from *Nrl*^−/−^ mice, based on the expression of cone markers (Fig. S4A). However, *Nrl*^−/−^ and wildtype cones are not identical because the former express rod arrestin, have shortened OS, ONL abnormalities, synapse onto rod bipolar cells^19^,^22^,^23^, and have a reduced cone flicker response (Fig. S4D). Thus, we will refer to photoreceptors from *Nrl*^−/−^;*Ccdc136*^*GFP*/+^ retinas as cod-GFP cells. Postnatal cod-GFP retinas were dissociated and analyzed for expression of GFP and retina cell type specific markers. At P1 and P6, all of the GFP^+^ cells co-expressed Crx and low to undetectable co-expression of proliferation (Ki67) and progenitor (Vsx2) markers (Fig. 4), which is consistent with a cone precursor cell identity. PNA expression was significantly increased in cods at P6 compared to P1 (% PNA in GFP^+^ cohort: P1, 19% ± 0.01; P6, 62% ± 2.6) However, postnatal cods were not fully mature, based on the absence of cone opsin and cone arrestin expression at P1 (data not shown). At this stage the GFP^+^ cells were not rod bipolar cells, as IHC analysis of cod-GFP retinas confirmed that these cells develop after P6 (Fig. S6) and lack of co-expression of the rod bipolar cell marker (PKC) in postnatal dissociates (Fig. 4a). We also noted that there was a lag between Crx and GFP expression, because the proportion of Crx^+^ cells that expressed GFP increased as a function of postnatal development (% GFP in Crx population: P1, 63% ± 0.02; P6, 85% ± 0.03; Adult approx. 90%). This observation suggests that induction of the *Ccdc136*^*GFP*^ reporter occurs after Crx expression is initiated in the cone differentiation cascade.

### Transplantation of GFP^+^-Cod precursors into adult recipient retinas

**Figure 5:**
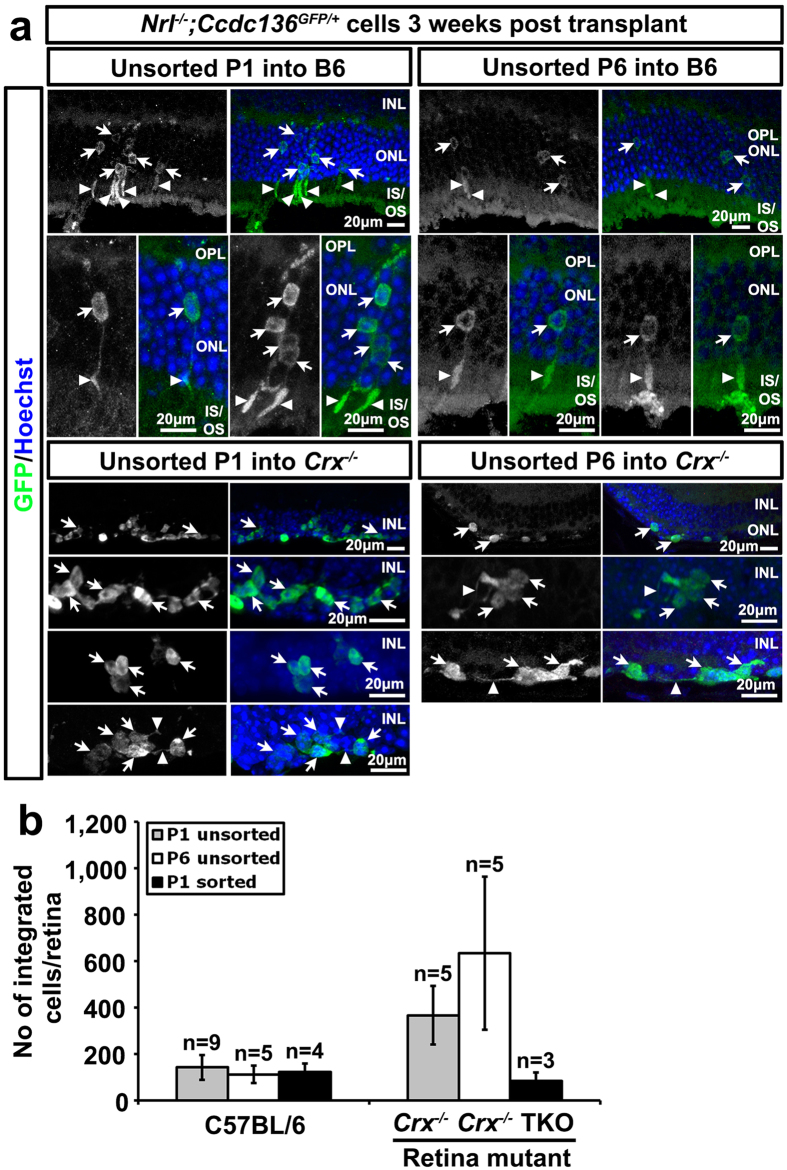
Integration of *Nrl*^−/−^*;Ccdc136*^*GFP*/+^ cells into adult C57Bl/6 (B6), *Crx*^−/−^ or *Gnat1*^−/−^*;Cnga3*^−/−^*;Opn4*^−/−^ (TKO) eyes. (**a**) Images of unsorted P1 or unsorted P6 transplanted cells integrated into indicated retinas. Arrows indicate GFP^+^ cell body, arrowheads indicate processes. INL, inner nuclear layer; IS/OS, inner segments/ outer segments; ONL, outer nuclear layer; OPL, outer plexiform layer. (**b**) Number of integrated *Nrl*^−/−^*;Ccdc136*^*GFP*/+^ cells in each recipient eye. Bars represent mean + SEM. Values include integrated cells from successful transplantations only.

The early and sustained reporter gene expression in cone precursor cells in the *Ccdc136*^*GFP*^ mice makes this mouse strain an ideal donor line to investigate the feasibility of cone engraftment into recipient retinas. Rod photoreceptor precursors capable of integrating in the adult retina are present in mixed dissociates isolated from P1–P7 donors^4^, with a subsequent study using donor rods ranging in age from P4–P6 to optimize engraftment^3^. While cods differentiate in the same temporal window as wildtype rods^19^ their integration potential had not been reported at the time these experiments were initiated. Therefore, we compared integration as a function of differentiation by transplanting dissociated retinal cells from cod-GFP donor mice at P1 (less mature based on low PNA expression) and P6 (more mature, based on higher PNA levels) (Fig. 4b) into adult wildtype retinas. To assess cell integration in the context of retinal degeneration we compared transplantation in mouse models of retinal disease. Photoreceptors in *Crx*^−/−^ mice are non-functional with extensive degeneration of the ONL at 4–6 months^24^, the age of the *Crx*^−/−^ recipient mice this study. As a second degeneration model, we used mice mutant for the combination of rod transducin (*Gnat1*^−/−^), cone cyclic nucleotide-gated channel (*Cnga3*^−/−^), and melanopsin (*Opn4*^−/−^) (triple knockout, TKO), which lack all light perception but maintain an ONL when they were transplanted at 4 months^25^. There was no significant difference in the integration rates of P1 and P6 cod-GFP cells into wildtype and mutant retinas, with both ranging from 100 to 600 cells per eye three weeks after transplantation (Fig. 5b), and comparable to control transplantations using un-enriched rod-GFP donor cells (Fig. S7). However, we did note an inverse relationship between donor age and the morphology of integrated cods in wildtype retinas. Integrated P1 cod-GFP cells were frequently found in clusters in the host ONL with the majority of cells adopting a photoreceptor-like morphology with apical and basal processes (Fig. 5a). In contrast, integrated P6 cod-GFP cells often failed to adopt photoreceptor morphology, remaining as single GFP^+^ cell bodies that lacked apical or basal processes (Fig. 5a). FACS enrichment of cells has been shown to increase rod integration^3^, however, this approach did not improve integration rates of P1 cods into wildtype and TKO retinal degeneration models (Fig. 5c and S8).

**Figure 6:**
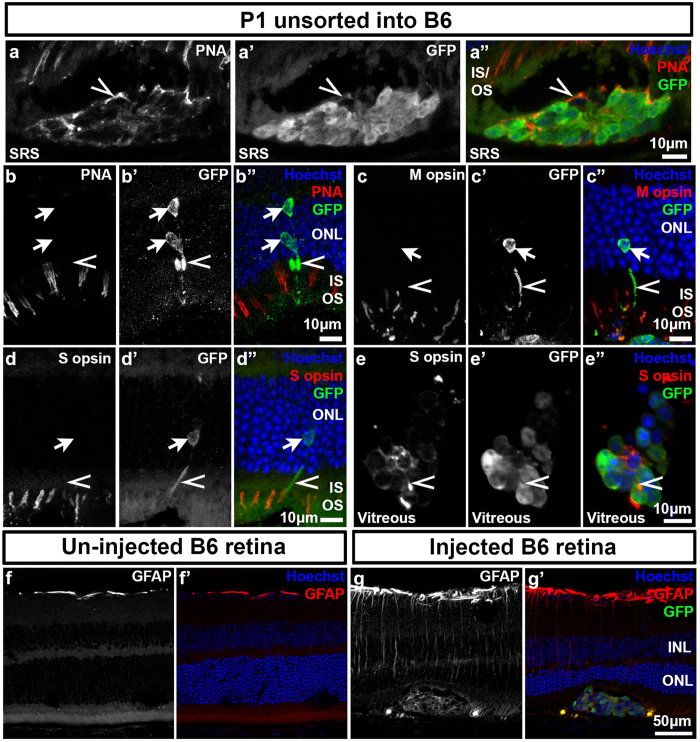
Differentiation of unsorted P1 *Nrl*^−/−^*;Ccdc136*^*GFP*/+^ cells after transplantation to adult C57Bl/6 (B6) retinas. (**a**–**e**) Transplanted GFP^+^ cells integrate into the host ONL and do not co-localize with the cone markers peanut agglutinin (PNA) (**b**), M opsin (**c**) or S opsin (**d**). Un-integrated cells in the subretinal space or in the vitreous express PNA (**a**) and S opsin (**e**). Arrows indicate cell body, arrowheads indicate processes. (**f**–**g**) Expression of the gliotic marker GFAP in a control un-injected B6 retina (**f**) and an injected B6 retina (**g**). INL, inner nuclear layer; IS/OS, inner segments/ outer segments; ONL, outer nuclear layer; SRS, subretinal space.

Despite adopting photoreceptor-like morphology, integrated P1 cod-GFP cells failed to express many mature cone markers, including PNA, S and M opsin, and cone arrestin (Fig. 6a and S9A). In contrast, expression of these markers was detectable in the un-integrated cells located in the subretinal space and in the vitreous (Fig. 6a,e), indicating that cells within the donor cod-GFP cell population have the potential to differentiate and can do so within 3 weeks. The differentiation kinetics for integrated rods is slower relative to cells that mature *in situ* or the subretinal space^26^ suggesting that some feature of the adult retinal environment is not conducive to timely maturation. One possibility is gliosis, which is induced by the surgical retinal detachment, and has been shown to correlate with reduced integration in mouse models of RP^5^. Consistent with this possibility, we detected gliosis localized to the site of retinal detachment and cell transplantation (Fig. 6b). Taken together our data suggests that there is an inverse relationship between donor age and ability of transplanted cods to adopt a photoreceptor-like morphology. Moreover, the maturation of GFP^+^ cells in cod-transplanted retinas is delayed relative to un-integrated cells and is associated with an injury response at the site of cell injection.

Because we could not detect cone marker expression in GFP^+^ cells located in the ONL of cod transplanted mice, we also considered the possibility that these cells represented a fusion event with host cells, most likely rod photoreceptors given their abundance relative to cones. GFP^+^ cells had single nuclei, which argues against simple cell fusion, and is consistent with findings from other groups^4^,^27^. Alternatively, the GFP^+^ signal in the host ONL could result from transfer of GFP from donor cells in the subretinal space to the OS of host photoreceptors. However, we observed integrated GFP^+^ cells in cod-transplanted *Crx*^−/−^ retinas, which lack OS (Fig. 5a). Moreover, there were many examples where the GFP signal of integrated cells extended only as far as the IS, which would not be consistent with retrograde flow of GFP from donor cells to host OS (Fig. 6a). We did find, however, examples where the nuclear morphology of integrated GFP^+^ cells matched the host rather than the donor cells. For instance, we observed GFP^+^ cells in the ONL of wildtype retinas from cod transplantations that had rod-like heterochromatin pattern (condensed with a central clump of heterochromatin) rather than cod-like (paler with multiple irregular clumps of heterochromatin) (Fig. S9A). However, we also observed integrated cods with a cod-like nuclear morphology (Fig. S9B). In summary, because of the absence of mature cone marker expression and the rod-like nuclear morphology of integrated GFP^+^ cells in cod-transplanted retinas we cannot entirely rule out the possibility that some of the integrated cells arise from fusion with host cells.

### Wildtype cone precursor transplantation

**Figure 7:**
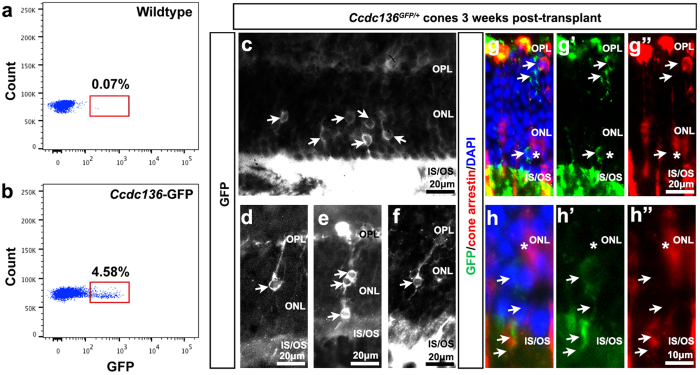
Integration of FACS sorted E17.5 *Nrl*^+/+^*;Ccdc136*^*GFP*/+^ cells into adult C57Bl/6 (B6) recipient eyes. (**a**,**b**) FACS profiles of dissociated E17.5 wildtype (**a**) and *Nrl*^+/+−^*;Ccdc136*^*GFP*/+^ (**b**). Viable cells were isolated based on GFP expression. (**c**–**f**) Examples of integrated GFP^+^ cells in the ONL, some showing polarized morphologies. (**g**-**g”**, **h**-**h”**) Co-labeling of sections with cone arrestin and GFP, demonstrating that GFP^+^ outer segments are present, but they fail to initiate cone arrestin expression. IS, inner segment; OS, outer segment; ONL, outer nuclear layer; OPL, outer plexiform layer.

We next took advantage of the *Ccdc136*^*GFP*^ reporter line to investigate integration of endogenous cone photoreceptors. Because of the low frequency of cones on a wildtype background, we modified our transplantation protocol by first FACS enriching for GFP^+^ cells and reducing the number of cells transplanted (approximately 10,000 cells injected/eye). We chose E17 donor cells, as we reasoned the cones at this stage would be similar in maturation to P1 cod-GFP cells, with low to undetectable levels of mature cone marker expression^12^. Three weeks post transplantation we observed integrated GFP^+^ cells in the ONL, several examples of GFP^+^ cells with apical and basal processes with the number of integrated cells ranging from 40 to 170 (n = 5 eyes), representing approximately 1% of injected cells. Similar to transplanted cod-GFP cells, transplanted cones were undifferentiated, based on the absence of cone arrestin expression (Fig. 7). These results show that this reporter line can be used for prospective enrichment of endogenous cones and that these cones can integrate into wildtype adult recipients.

## Discussion

Here we characterize a novel GFP reporter mouse line that marks developing and mature cone photoreceptors and rod bipolar cells. The induction of the reporter gene is observed as early as E13.5 in postmitotic cone photoreceptors located at the apical side of the neuroepithelium, and is maintained throughout cone differentiation to maturity. Consistent with expression in cones, the GFP reporter is expressed by the majority of photoreceptors in the cone-only Nrl^−/−^ background. We show that GFP-tagged endogenous cones and cods can integrate in the adult wildtype and degenerative retina and exhibit photoreceptor morphology. This reporter line will be useful for early analysis of cone cell development and for cone transplantation platforms.

Lineage specific fluorescent reporter lines are advantageous for monitoring cell development *in situ* and prospective cell enrichment, particularly if their temporal expression pattern is broader and does not affect cell survival compared with currently available lineage markers. The *Ccdc136*^*GFP*^ mouse line described here fulfills these criteria, as it is expressed earlier in cone development than other lineage markers and the cytoplasmic localization of GFP can be used to monitor the morphological maturation of cones. In contrast to the OPN1LW-GFP reporter line^10^, expression of the *Ccdc136*^*GFP*^ allele captures a larger number of embryonic cones and does not induce cone degeneration. One disadvantage of this line as a cone reporter is its weak expression in M cones, making it primarily an S and S/M cone reporter line. The expression of the reporter in both cones and rod bipolar cells could also be problematic for cell enrichment based on GFP alone at adult stages. However, because the GFP expression in these lineages is spatially and temporally segregated, this line will still be useful for prospective enrichment of endogenous cones or rod bipolar cells, at early stages of development and for *in situ* analysis of cone and rod bipolar cell differentiation.

Ccdc136 is predicted to be a single-pass membrane protein with coiled-coil domains, but lacks significant homology to other coiled-coil domain proteins. Although Ccdc136 has been identified as a candidate interacting protein in several yeast 2 hybrid screens, these studies have not shed light on its function because the baits belong to many distinct functional protein classes including RNA metabolism^28^, neuronal activity^29^, neural degeneration^30^ and signal transduction^31^. The lack of a retinal phenotype in *Ccdc136*^*GFP/GFP*^ mice suggests that this gene could function redundantly with other proteins in retinal cells, which is consistent with the expression of *Ccdc66* in rod photoreceptor OS^32^. However, it is also possible, that low levels of the Ccdc136 protein are still produced in *Ccdc136*^*GFP/GFP*^ retina, based on our evidence that the gene trap sequences can be spliced out to generate wildtype transcripts from this allele.

Here we have investigated the feasibility of using this reporter line to track transplanted cone photoreceptors. We show that GFP-marked cods and cones can integrate in the adult retina and undergo morphological differentiation. Though it has been previously reported that the cell bodies of integrated embryonic Crx-GFP marked cones were located on the apical side of the ONL^9^, we observed cell bodies of integrated Ccdc136^GFP^-tagged cods and cones at all levels of the ONL. In the case of cods, their nuclear location could reflect their rod-like properties (see below). This difference in nuclear positioning between Crx-GFP and Ccdc136^GFP^ embryonic cones could be explained by an influence of co-transplanted rods in the Crx-GFP model.

Because integrated *Ccdc136*^*GFP*^-tagged cods and cones did not mature fully in terms of expression of the phototransduction cascade proteins, it is unlikely they would mediate vision rescue in blind mice. Moreover, the numbers of integrated cods in our study was considerably lower than the number of functional photoreceptors required to measure an ERG response^3^. In contrast, Santos-Ferreira *et al.*^33^, reported differentiation and functional cone integration in retinas of mice transplanted with cods isolated from the retinas of mice expressing a ubiquitous GFP reporter. While we cannot compare functional outcomes (since we did not test function in cod-transplanted mice) it is difficult to reconcile the differences in cod differentiation between these two studies. Since the GFP reporters used in both studies do not interfere with cone function or development, a more plausible explanation is differences in the length of time post transplantation before analysis, which in our study was 3 weeks versus 4 weeks as reported by Santos-Ferreira *et al.*^33^. The differentiation of integrated rod precursor cells is also delayed relative to rods developing *in situ*^26^ and suggests that this process is affected by some property of the adult retina, possibly the lack of positive cues or the presence of inhibitory cues. Consistent with the latter possibility, we observed gliosis, which is known to inhibit cell integration in the retina^5^,^34^, at the site of injection-induced subretinal detachment.

The lack of mature cone marker expression in the integrated GFP^+^ cods and cones prompted us to consider whether some of these cells result from fusion with endogenous host photoreceptors. Simple cellular fusion is an unlikely explanation as the integrated cells had single nuclei and fusion events have not been reported in rod and cod transplantation studies where this possibility was tested using dual reporter systems^4^,^27^,^33^. Moreover, cell fusion is difficult to reconcile with the evidence for improved retinal function following transplantation of rod and cod precursor cells in blind mouse models where the host ONL is relatively intact^3^,^4^,^33^. Moreover, we could detect integrated GFP^+^ cods with processes (albeit misaligned) in degenerated *Crx*^−/−^ retinas, demonstrating that intact host photoreceptors are not required to detect transplanted cod-GFP cells. We did, however, observe GFP^+^ cells in the host ONL that had host instead of donor cell nuclear morphology. Whether this nuclear mismatch represents cell fusion or donor cells changing nuclear morphology in their new environment will require further investigation.

While post mitotic rod precursors have the best integration potential, the molecular basis for this property is not well understood. In our study the integration rates of P1 and P6 donor cods were comparable to those reported for unsorted P3–P6 rod precursors^3^,^4^ and sorted cods^33^ transplanted into wildtype retinas. However, in contrast to other studies^4^,^27^,^33^, we did not observe an increase in integration rates with older (P6) compared with younger (P1) donor cods, nor did we find that FACS enrichment improved integration rates. Donor cells at P6 are still within the range of optimal integration efficiency, as integration rates for rods and cods ranging in age from P2–P7 are not significantly different^4^,^33^. However, we were at the very low end of the spectrum for integration, which could have precluded seeing age-related differences. We did, however, observe better morphology of integrated P1 compared with P6 donor cells and this difference correlates with less PNA expression in donor cods at P1. While a similar relationship between integrated cell morphology and donor photoreceptor age has not been reported, rod precursors with a cell surface marker profile characteristic of less differentiated rods exhibit better integration compared with the global rod precursor population at the same stage^35^. In future experiments, it will be important to combine cell surface and cone-differentiation markers together with the Ccdc136^GFP^ reporter to stratify the cone precursor pool and identify those with the best morphological and functional integration.

In contrast to integrated cods and cones, expression of mature cone markers was readily observed in un-integrated cells located in the subretinal space and the vitreous, which is consistent with the differentiation of un-integrated rod photoreceptor precursors in other studies^26^,^27^. In contrast to Eberle *et al.*^36^, we did not observe an obvious difference in the levels of PNA staining in cells located in the subretinal space and vitreous in animals transplanted with unsorted cods. Thus, it is possible that differentiation signals from the co-transplanted non-photoreceptor cells compensate for pro-differentiation signals postulated to be present in the subretinal space^36^.

*Nrl*^−/−^ cods exhibit some differences from wildtype cone photoreceptors^19^,^22^,^23^; thus, it could be argued that cods are not a viable surrogate for cone transplantation studies. However, it is not entirely clear whether these defects are cell autonomous to cods versus non-cell autonomous effects associated with an all-cone retina. Indeed, defects in outer limiting membrane^37^ and chromophore production from the RPE^38^ affect the ONL morphology and organization in the *Nrl*^−/−^ retina. Consistent with an environmental contribution to cod dysmorphology, transplanted cods exhibit better morphology compared to cods that differentiate *in situ* in the *Nrl*^−/−^ retina (this study and^33^) consistent with the possibility that aberrant OS development in the Nrl^−/−^ retina is secondary to the absence of rods. It will be important to determine whether other abnormalities in cods are rescued following transplantation to a wildtype environment.

## Materials and Methods

### Animals and genotyping

All experiments were approved by the University of Ottawa Animal Care Ethics Committee and adhered to the guidelines of the Canadian Council on Animal Care. Animals were maintained under standard laboratory conditions and all procedures were performed in conformity with the University of Ottawa Animal Care and Veterinary Service, and the University of Calgary Animal Care Committee (protocol #AC11–0053) in compliance with the Guidelines of the Canadian Council of Animal Care. Wildtype C57BL/6 mice were obtained from Charles River. The following transgenic mouse strains used in this study were maintained as homozygous lines: *Nrl*-EGFP rod photoreceptor reporter^7^, *Nrl*^−/−^ cone-only^19^, *Crx*^−/−^ cone and rod dysfunction and degeneration^24^, *Gnat1*^−/−^*;CNGA3*^−/−^*;Opn4*^−/−^ lacking all rod, cone and photosensitive ganglion cell function (triple-knockout, TKO^25^) and *Ccdc136*^*GFP/GFP*^ cone-GFP reporter. The *Ccdc136*^*GFP*^ transgenic mice were generated by blastocyst injection of the CMHD-GT_373F8-3 ES cell line. Gene trap insertion was confirmed by PCR amplification and sequencing using exon and vector specific primer pairs. The genomic/vector junction was determined to be at chr6: 29, 401, 913. For genotyping genomic DNA was isolated by incubating tissue from an ear clip in 300 μl alkaline lysis buffer (25 mM NaOH, 0.2 mM EDTA pH 8.0) for 60 minutes at 95 °C. Samples were neutralized with 300 μl neutralization reagent (40 mM Tris-HCl) and genotyped by PCR using primer sets indicated in Supplemental Table 1.

### Tissue preparation, immunohistochemistry and *in situ* hybridization

Eyes were harvested at various stages beginning at embryonic day (E) 13.5 to adult. Mice younger than postnatal day (P) 10 were culled and entire heads with eyes were collected. Mice older than P10 were perfused with 4% paraformaldehyde (PFA) and eyes were marked on their corneas for dorsal orientation with a silver nitrate stick before enucleating. Tissues were fixed in 4% PFA overnight then cryoprotected overnight in 30% sucrose in phosphate buffered saline (PBS) (0.14 M NaCl, 2.5 mM KCl, 0.2 M Na2HPO4, 0.2 M KH2PO4). After sinking in sucrose, tissues were equilibrated in 50:50 30% sucrose: OCT (Tissue-Tek) for 1–2 hours, followed by orienting and embedding in plastic molds in sucrose:OCT and freezing. Samples were stored at −80 °C. Tissue was sectioned at 12 μm onto Superfrost Plus slides (Fisher Scientific) on a Leica cryostat, air-dried for 1 to 2 hours and stored at −20 °C with desiccant. Retinal sections and dissociated cells were permeabilized with 70% ethanol for 5 minutes then washed in PBS for 20 minutes. Following an antigen retrieval step (for Crx, PKC and Ki67 antibodies), sections were blocked with 50 mM Tris buffer (pH 7.4), 10 mM lysine, 145 mM NaCl and 1% BSA (TBLS; blocking solution) for 1 hour at room temperature. Primary antibodies (listed in Supplemental Table 2) were diluted in TBLS for overnight staining of retina sections at 4 °C. After several washes with PBS, sections were incubated with fluorescent secondary antibody diluted in TBLS for 1 hour at room temperature in the dark. Nuclei were counterstained with fluorescent DNA-binding dye, Hoechst (Life Technologies). For IHC analysis of dissociated cells, 10 μl of cell suspension was streaked onto the slides and was incubated at room temperature for 5 minutes followed by 40 minutes at 37 °C in a humidified box. Cells were fixed with 4% PFA for 15 minutes. After several washes in PBS, cells were stained as described above. Slides were washed and glass coverslips were mounted with DAKO mounting media. *In situ* hybridization was carried out as previously reported^39^ and detailed protocols are available upon request.

### Retinal dissociation, Fluorescence activated cell sorting (FACS), and subretinal injection of postnatal Cods and embryonic cones

Detailed methods are available in the Supplementary information. Briefly, 200,000 papain (postnatal unsorted and FACS enriched *Nrl*^−/−^*;Ccdc136*^*GFP*/+^) and 10,000 trypsin (embryonic FACS enriched *Ccdc136*^*GFP*/+^) dissociated cells were transplanted to the subretinal space of adult recipient C57Bl/6, *Crx*^−/−^ and TKO mice (1–6 months old). Donor cell integration was assessed three weeks after transplantation.

### Imaging, cell counts and statistical analysis

Fluorescent images were captured using an Axioimager M1 (Carl Zeiss, Inc.) or an Axioimager 2 (Carl Zeiss, Inc.). Confocal images were acquired using a FluoView 1000 confocal microscope (Olympus), a LSM 700 (Carl Zeiss) or a LSM 780 (Carl Zeiss, Inc.). Wildtype and mutant retinas were imaged with identical exposure times at each magnification. Images were processed using Photoshop CS4 (Adobe) and any adjustments were made to the entire image and equally for each genotype. Quantification of dissociated cells was performed by counting marker+ cells and dividing by the total number of nuclei (5 images taken per stain with a minimum of 200 cells counted). Transplanted eyes were harvested three weeks post surgery. Retina cryosections, prepared as described above, were stained with anti-GFP and cell type specific antibodies (Supplemental Table 1). Cells were scored as integrated if the entire cell body was located in the host outer nuclear layer (ONL), or, in the case of *Crx*^−/−^ recipients, in or adjacent to the INL. The average number of integrated cells per eye was determined by counting all integrated GFP^+^ cells in alternate sections of each eye and multiplying that number by two. Only animals with successful transplantations, where GFP^+^ cells were located in the subretinal space, were included in the quantitative analysis. If there was evidence of intravitreal cell delivery, bleeding into the eye or if no GFP^+^ cells were detected in the subretinal space, these animals were considered as “failed transplants” and excluded from further analysis. All data are presented as mean ± SEM. Statistical significance was evaluated using a two-tailed paired *t*-test or 2-way ANOVA.

## Additional Information

**How to cite this article**: Smiley, S. *et al.* Establishment of a cone photoreceptor transplantation platform based on a novel cone-GFP reporter mouse line. *Sci. Rep.* **6**, 22867; doi: 10.1038/srep22867 (2016).

## Supplementary Material

Supplementary Information
