# Corrigendum: Establishment of a cone photoreceptor transplantation platform based on a novel cone-GFP reporter mouse line

**DOI:** 10.1038/srep24012

**Published:** 2016-04-22

**Authors:** Sheila Smiley, Philip E. Nickerson, Lacrimioara Comanita, Narsis Daftarian, Ahmed El-Sehemy, En Leh Samuel Tsai, Stuart Matan-Lithwick, Keqin Yan, Sherry Thurig, Yacine Touahri, Rajiv Dixit, Tooka Aavani, Yves De Repentigny, Adam Baker, Catherine Tsilfidis, Jeff Biernaskie, Yves Sauvé, Carol Schuurmans, Rashmi Kothary, Alan J. Mears, Valerie A. Wallace

Scientific Reports 6: Article number: 22867 10.1038/srep22867; published online: 03112016; updated: 04222016

The original version of this Article contained a typographical error in the spelling of the author Yves De Repentigny, which was incorrectly given as Yves De Repentingy. This has now been corrected in the PDF and HTML versions of the Article.

