# Supplementary material for: Establishment of a cone photoreceptor transplantation platform based on a novel cone-GFP reporter mouse line
Source: Sci Rep. 2016 Mar 11;6:22867. doi: 10.1038/srep22867 (PMC4786810; doi:10.1038/srep22867)

Running title: Cone photoreceptor reporter mouse strain

Establishment of a cone photoreceptor transplantation platform based on a novel cone-GFP reporter mouse line

Sheila Smiley[1,4,6], Philip E. Nickerson[6], Lacrimioara Comanita[6], Narsis Daftarian[8], Ahmed El-Sehemy[6,7], En Leh Samuel Tsai[6,7], Stuart Matan-Lithwick[6,7], Keqin Yan[1], Sherry Thurig[1], Yacine Touahri[8], Rajiv Dixit[8], Tooka Aavani[8], Yves De Repentingy[1], Adam Baker[1], Catherine Tsilfidis[1,2,4], Jeff Biernaskie[9], Yves Sauve[10], Carol Schuurmans[8], Rashmi Kothary[1,4,5], Alan J. Mears[1,3] and Valerie A. Wallace*[1,2,3,6,7]

[1]Ottawa Hospital Research Institute, 501 Smyth Road, Ottawa, Ontario, K1H 8L6, Canada

[2]Departments of Ophthalmology, [3]Biochemistry, Microbiology and Immunology, and

[4]Cellular and Molecular Medicine, University of Ottawa

[5]Department of Medicine, University of Ottawa

[6]Division of Vision Science, Krembil Research Institute, University Health Network and Department of Ophthalmology and Vision Science

[7]Department of Laboratory Medicine and Pathobiology, University of Toronto

[8]Biochemistry and Molecular Biology, [9]Comparative Biology and Experimental Medicine, Hotchkiss Brain Institute and Alberta Children's Hospital Research Institute, University of Calgary, 3330 Hospital Drive NW, Calgary, Alberta Canada, T2N 4N1

[10] Department of Ophthalmology and Visual Sciences, University of Alberta

*Address for correspondence:
Valerie Wallace, PhD
KRI, 60 Leonard St. Toronto ON M5T 2S8
Phone: 416-603-5800 x 7378
vwallace@uhnresearch.ca

Key Words: retina, transplantation, cone photoreceptor, GFP, Nrl

Supplemental Information

Methods

Electroretinography

Electroretinogram (ERG) recordings were carried out on *Ccdc136*$^{+/+}$ (n=12), *Ccdc136*$^{GFP/GFP}$ (n=13), *Nrl*$^{-/-}$;*Ccdc136*$^{+/+}$ (n=6) and *Nrl*$^{-/-}$;*Ccdc136*$^{GFP/+}$ (n=5) mice. In brief, the mice were dark-adapted for 1 hour before anesthesia with a mixture of ketamine (62.5 mg/kg IP) and xylazine (12.5 mg/kg IP) and the pupils dilated with 1% tropicamide. Body temperature was monitored with a rectal thermometer and maintained at 38°C with a homeothermic electric blanket. Simultaneous bilateral recording was achieved with active gold loop electrodes (placed on each cornea) and a subdermal platinum reference electrode (placed on the scruff); a subdermal ground platinum electrode was placed on the tail. Light stimulation (10μs flashes), signal amplification (0.3–300-Hz bandpass), and data acquisition were provided by an electrophysiology system (Espion E2; Diagnosys LLC, Littleton, MA). For each animal, only one eye was considered for statistical comparisons. It corresponded to the eye associated with the dark-adapted b-wave of maximum amplitude.

Retinal dissociation and subretinal injection of postnatal Cods and embryonic cones

For transplantation experiments, retinas from *Nrl*$^{-/-}$;*Ccdc136*$^{GFP/+}$ and *Nrl*-EGFP mice were dissociated with papain (Worthington Biochemical, UK) according to the manufacturer's directions. Cells were suspended in 5% fetal bovine serum (FBS; Sigma, Cat# F1051) in Ca^{2+}/Mg^{2+} free PBS and transferred to FACS tubes (BD Falcon 12 x 75 mm Tube with Cell Strainer Cap) filtering out any cellular debris. GFP^{+} cells were sorted

using a MoFlo (Beckman Coulter) cell sorter system, collecting 2,000,000 cells in 20% FBS in Earl's Balanced Salt Solution (EBSS). Sorted and unsorted cells were re-suspended at 200,000 cells/μL in EBSS and kept on ice prior to transplantation. Adult recipient C57Bl/6, *Crx*$^{-/-}$ and *Gnat1*$^{-/-}$; *Cnga3*$^{-/-}$; *Opn4*$^{-/-}$ (triple knockout, TKO) mice (1-6 months old) were anesthetized by isoflurane gas inhalation. Eyes were dilated using 1% tropicamide (Mydriacyl, Alcon) drops and 0.5% proparacaine hydrochloride (Alcaine, Alcon) drops were administered as a local anesthetic. A 0.2% hypromellose gel (Genteal, Novartis) was applied to maintain lubrication of the corneas. For sub-retinal injection via the intravitreal route, the left eye of the recipient mouse was gently prolapsed and a scleral puncture was made on the dorsal side directly posterior to the limbus with a V-lance knife. Injections were carried out using a nano-injector (Harvard Apparatus) and performed using a surgical microscope. A blunt 34 gauge needle (Hamilton) was inserted tangentially through the sclera and across the vitreous to the opposite ventral side and moved into the subretinal space until there was resistance. The needle was retracted slightly and 1 μL of cell suspension was injected over 30 seconds. The needle was held in place after injection for 10-20 seconds to allow for equilibration of pressure and then retracted slowly. After injection, a coverslip coated with 0.2% hypromellose gel was placed on top of the cornea to allow for visualization of the back of the eye to ensure subretinal delivery of cells. Right eyes served as untreated controls. For subretinal injection of embryonic cones adult recipient mice were treated with a single intravitreal dose of L2/DL amino-adipic acid (AAA; Sigma; 66.66 μg/μL in 1xPBS) 72 hr prior to the subretinal injection in the same eye. AAA was used to disrupt the outer limiting membrane to increase donor cell integration[1]. For cell transplantation a blunt 30 gauge

hypodermic needle (VWR) was used to create a superficial nick on the sclera at the posterior region of the superotemporal side of the globe. Next, a sharp 36 gauge needle was used to create a nick in the cornea to reduce the intraocular pressure. A blunt 32 gauge needle then was inserted through the scleral nick and guided tangentially under the sclera into the subretinal space and 1 μL of cell suspension was injected slowly over 30 seconds. The needle was held in place after injection for 10-20 seconds to allow for equilibration of pressure and then slowly retracted. A coverslip coated with 0.2% hypromellose gel was placed on top of the cornea to confirm the presence of a localized retinal detachment.

Fluorescence activated cell sorting (FACS) of embryonic cones

E17.5 embryos from *Nrl*$^{+/-}$;*Ccdc136*$^{GFP/+}$ pregnant females were dissected in cold phosphate buffered saline (PBS) and neural retinas separated from the RPE. Pooled neural retinas (from 6-8 embryos/litter) were placed in ~100 uL of cold $Ca^{2+/}Mg^{2+/}$ free PBS. Tissue was dissociated by trypsinizing in 2 mL of 0.125% Trypsin (Sigma, Cat# T1005-1G) in Ca^{2+}/Mg^{2+} free PBS pre-warmed to 37°C for 5-10 min while shaking. To stop the trypsinization, 3 mL of 20% FBS in PBS was added and the tissue was triturated by pipetting up and down ~40 times with a 5 mL pipet. Cells were then pelleted by centrifuging at 520 x g for 5 min at 4°C, and washed in 5ml of ice-cold Ca^{2+}/Mg^{2+} free PBS containing 0.1% BSA and 5mM EDTA (Solution 1), and re-suspended in 1 mL Solution 1. 0.3 mL of the cell suspension was removed as a negative – no stain control while the remaining cells were mixed with a viability dye (1:1000, Viability Dye eFluor 780 from eBioscience) and stained for 20 min in the dark on ice. After staining, 9 mL of

Solution 1 was added to the cells. Cells were re-pelleted and resuspended in 5-10 mL of Solution 1 two more times to wash away unbound stain. Cells were resuspended in 0.5 mL of Solution 1 (total volume = 0.3 mL) and transferred to FACS tubes (BD Falcon 12 x 75 mm Tube with Cell Strainer Cap), filtering out any cellular debris. GFP+ cells were FACS-sorted using a BD FACSAria™ III cell sorter system, collecting 100,000 *GFP* positive cells in approximately 10 mL. Cells were resuspended in Solution 1 at a concentration of 10,000 cells/μL.

References

1. West, E. L. *et al.* Pharmacological disruption of the outer limiting membrane leads to increased retinal integration of transplanted photoreceptor precursors. *Exp Eye Res* **86**, 601-611 (2008).

Supplemental Table 1 Primers sequences used for genotyping

**Primer**	**Sequence (5' - 3')**
Ccdc136 wildtype forward	CCGTGGTGGGGGTTGAATCCAA
Ccdc136 wildtype reverse	TGGCAAAGTCATGAAGGGACCACA
Ccdc136 GFP forward	CACATGAAGCAGCACGACTT
Ccdc136 GFP reverse	TGCTCAGGTAGTGGTTGTCG
Cnga3 wildtype forward	TCAACCGCCTCCTGAAGTTCTC
Cnga3 wildtype reverse	CCCACAGATAGTCAAACCACC
Cnga3 knockout forward	CTTAGGTTTCCTTGAGCAAGGC
Cnga3 knockout reverse	GCCTGCTCTTTACTGAAGGCTCT
Crx forward	AGGAGTTTGGCATTCCAGGG
Crx reverse	CGATGATCTCGTCGTGACCC
Gnat1 wildtype forward	CGAGTTCATTGCCATCATCTACG
Gnat1 wildtype reverse	ATACCCGAGTCCTTCCACAAGC
Gnat1 knockout forward	GAGGATTGGGAAGACAATAGCAG
Gnat1 knockout reverse	CACCAGCACCATGTCGTAAG
Nrl wildtype forward	GTGTTCCTTGGCTGGAAAGA
Nrl wildtype reverse	CTGTTCACTGTGGGCTTTCA
Nrl knockout forward	TGAATACAGGGACGACACCA
Nrl knockout reverse	GTTCTAATTCCATCAGAAGCTGAC
Opn4 wildtype forward	TTACTACTCAAGAAGATTGGTTCTCAG
Opn4 wildtype reverse	CATCCTAACATTACTAGATCCAGCTTT
Opn4 LacZ knockout forward	GCGATGGGTAACAGTCTTGG
Opn4 LacZ knockout reverse	CGGTAGTTCAGGCAGTTCAATC

Supplemental Table 2. Primary antibodies used in this study

Antibody	Cell type specificity	Species	Supplier
Anti-GFP		Goat	Rockland (600-101-215)
Anti-AP-1	Amacrine cells	Rabbit	DSHB (3B5-c)
Anti-Calbindin	Horizontal cells	Mouse	Sigma (C9848)
Anti-Cone arrestin	Cone	Rabbit	Millipore (AB15282)
Anti-Crx	Photoreceptors, Bipolar cells	Rabbit	Gift from C. Gregory-Evans
Anti-GFAP	Astrocytes, reactive Muller glia	Rabbit	Sigma (G9269)
Anti-Glutamine Synthetase	Muller glia	Rabbit	Abcam (ab49873)
Anti-Ki67	Cycling cells	Mouse	BD Pharmingen (550609)
Anti-M-Opsin	Cone OS	Rabbit	Millipore (AB5405)
Anti-S-Ospin	Cone OS	Rabbit	Millipore (AB5407)
Anti-Pax6	Amacrine, Ganglion cells	Rabbit	Life Technologies (42-6600)
Anti-PKC	Rod bipolar cells	Mouse	Abcam (ab31)
Anti-PDE6C	Cone OS	Rabbit	Thermo Scientific (PA5-26732)
PNA	Cone IS and OS	Lectin biotinylated	Vector (B-1075)
Anti-RBPMS	Ganglion cells	Rabbit	PhosphoSolutions (1830-RBPMS)
Anti- Recoverin	Photoreceptors	Rabbit	Millipore (AB5585)
Anti-Rhodopsin	Rod OS	Mouse	DSHB (B630)
Anti-RXRγ (Y-20)	Cone	Rabbit	Santa Cruz (sc-555)
Anti-Vsx2	Progenitors, bipolar cells	Sheep	Exalpha (X1179P)

Supplemental Figure Legends

Figure S1. Genomic organization of wildtype and gene-trapped *Ccdc136* alleles. (A) Mouse *Ccdc136* is located at 6qA3.3 on the positive strand, downstream of *Opn1sw*, found on the negative strand. (B) *Ccdc136*-GFP mutant allele, created by gene-trap. pUPA was inserted 2,303 bp into intron 2 of mouse *Ccdc136*. Two mRNAs are predicted. One includes *Ccdc136* exons 1 & 2 fused to IRES and GFP. The other consists of NeoR, floxed IRES sequences (blue), and *Ccdc136* exons 3 to 15. SA, Splice Acceptor; IRES, Internal Ribosome Entry Site; GFP, Green Fluorescent Protein; PA, Poly A; RNAPII Pro, RNA Polymerase II Promoter; Neo-R, Neomycin Resistance; Thin blue segments, LoxP sites; SD, Splice Donor. (C) RT-Q-PCR analysis of *Ccdc136* transcripts in retina of $Ccdc136^{+/+}$, $Ccdc136^{GFP/+}$, and $Ccdc136^{GFP/GFP}$ mice normalized to GAPDH. The 5' primer set spans exons 2-3, which would only amplify wildtype transcript. * $P < 0.05$, n=3.

Figure S2. Expression of retinal cell markers in *Ccdc136*-GFP retinas. (A) Expression of rhodopsin in adult $Ccdc136^{GFP/+}$ dissociated cells. GFP^{+} cells indicated by arrows. $Rhodopsin^{+}$ cells indicated by open arrowhead. (B) Expression of indicated markers in adult wildtype and adult $Ccdc136^{GFP/GFP}$ retinas. Glutamine synthetase (GS) labels Müller glia, RBPMS labels ganglion cells, calbindin labels horizontal cells, Pax 6 labels amacrine cells, rhodopsin labels the OS of rod photoreceptors, and Vsx2 labels bipolar cells. GCL, ganglion cell layer; INL, inner nuclear layer; ONL, outer nuclear layer; OS, outer segments.

Figure S3. Adult *Ccdc136*$^{GFP/GFP}$ cones exhibit persistent expression of mature cone markers throughout adulthood. (A) GFP^{+} cones in *Ccdc136*$^{GFP/GFP}$ retinas exhibit a ventral (higher)-to-dorsal (lower) gradient at 8 months and co-localize with peanut agglutinin (PNA, red) and cone arrestin (CAR, blue). (B) Wildtype dorsal and ventral PNA and CAR reference staining. (C) Quantification of cones expressing GFP, PNA, or CAR in *Ccdc136*$^{GFP/GFP}$ or wildtype mice at 3 and 8 months of age (n = 3 per group). Counts (means and 1.0 standard error) were performed in a 20x field at dorsal, central and ventral locations, and represent the total number cells per 300 µm of retina. Statistical significance assessed by paired t-test.

Figure S4. Characterization of *Nrl*$^{-/-}$*;Ccdc136*$^{GFP/+}$ retinas. (A) Expression of cone photoreceptor markers in adult *Nrl*$^{-/-}$*;Ccdc136*$^{GFP/+}$ retinas. Cone arrestin (CAr) is a pan cone marker. PNA labels cone outer segments. M opsin and S opsin label outer segments of M and S cones. Rhodopsin labels rod photoreceptor outer segments and is not expressed in the *Nrl*$^{-/-}$*;Ccdc136*$^{GFP/+}$ retina. INL, inner nuclear layer; ONL, outer nuclear layer; OS, outer segments. (B-D) Comparison of the electroretinograph (ERG) photopic (cone-dependent) responses in *Ccdc136*$^{+/+}$, *Ccdc136*$^{GFP/GFP}$, *Nrl*$^{-/-}$ and *Nrl*$^{-/-}$*;Ccdc136*$^{GFP/+}$ mice. Average values for each step are presented for photopic a waves (B), b waves (C), and flicker frequency series (D). *Ccdc136*$^{+/+}$ (n=12), *Ccdc136*$^{GFP/GFP}$ (n=13), *Nrl*$^{-/-}$*;Ccdc136*$^{+/+}$ (n=6) and *Nrl*$^{-/-}$*;Ccdc136*$^{GFP/+}$ (n=5). (E) Expression of M opsin (red) and S opsin (green) in retinal flatmounts from ERG analyzed mice. Statistical

significance between *Ccdc136*$^{+/+}$ and *Nrl*$^{-/-}$;*Ccdc136*$^{+/+}$ groups and *Ccdc136*$^{GFP/GFP}$ and *Nrl*$^{-/-}$;*Ccdc136*$^{GFP/GFP}$ groups assessed by 2-way ANOVA; n.s. not significant.

Figure S5. Central to peripheral developmental gradient of cones in postnatal day 4 (P4) *Ccdc136*$^{GFP/+}$ retinas. GFP$^+$ (green) cones in the central retina migrate basally in the ONL (left column). GFP^{low} cells are also seen in the ONL and apical INL. Absence of Ki67$^+$ (red) cells in the central retina indicates GFP$^+$ cells are post-mitotic. GFP$^+$ cones in the peripheral retina are located apically.

Figure S6. Expression of GFP in the INL of postnatal *Nrl*$^{-/-}$*;Ccdc136*$^{GFP/+}$ retinas. GFP$^+$ cells in the INL (arrows) begin to appear in P10 and P12 retinas. INL, inner nuclear layer; ONL, outer nuclear layer.

Figure S7. Integration of *Nrl*-EGFP cells into adult C57Bl/6 (B6), *Crx*$^{-/-}$ or *Nrl*$^{-/-}$ eyes. (A) Images of unsorted P4 transplanted cells integrated into indicated retinas. Arrows indicate GFP+ cell body, arrowheads indicate processes. GCL, ganglion cell layer; INL, inner nuclear layer; IS/OS, inner segments/outer segments; ONL, outer nuclear layer. (B) Number of integrated *Nrl*-EGFP cells in each recipient eye. Bars represent mean. Values include integrated cells from successful transplantations only.

Figure S8. Transplantation of FACS sorted postnatal day 1 (P1) *Nrl*$^{-/-}$*;Ccdc136*$^{GFP/+}$ cells into adult C57Bl/6 (B6) and *Gnat1*$^{-/-}$*;Cnga3*$^{-/-}$*;Opn4*$^{-/-}$ (triple knockout, TKO) eyes.

Arrows indicate GFP^+ cell body, arrowheads indicate processes. IS/OS, inner segments/ outer segments; ONL, outer nuclear layer.

Figure S9. Nuclear morphology of transplanted $Nrl^{-/-};Ccdc136^{GFP/+}$ cells into adult C57Bl/6 (B6) eyes. (A) i. GFP^+ integrated cell shows rod photoreceptor nuclear morphology with condensed heterochromatin. ii. Compare to Cone arrestin $(CAr)^+$ cone photoreceptor nuclear morphology with irregular heterochromatin. iii. Transplanted GFP^+ cells in the subretinal space show cone photoreceptor nuclear morphology. (B) GFP^+ integrated cell shows cone photoreceptor nuclear morphology. Arrows indicate cell body. IS/OS, inner segments/ outer segments; ONL, outer nuclear layer.

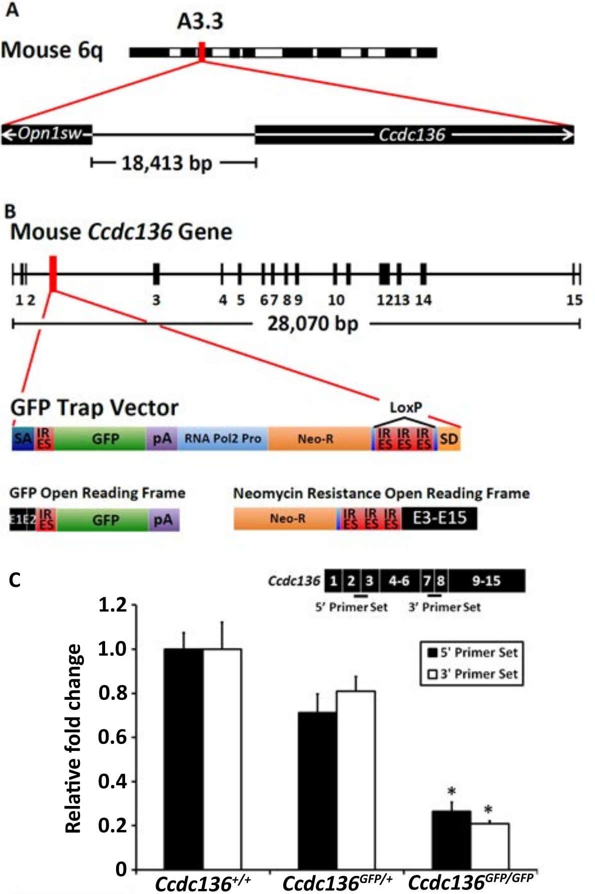

Average CT	$Ccdc136^{+/+}$	$Ccdc136^{GFP/+}$	$Ccdc136^{GFP/GFP}$
GAPDH	19.65	19.41	19.93
5′ *Ccdc136*	24.14	24.40	26.34
3′ *Ccdc136*	26.10	26.18	28.64

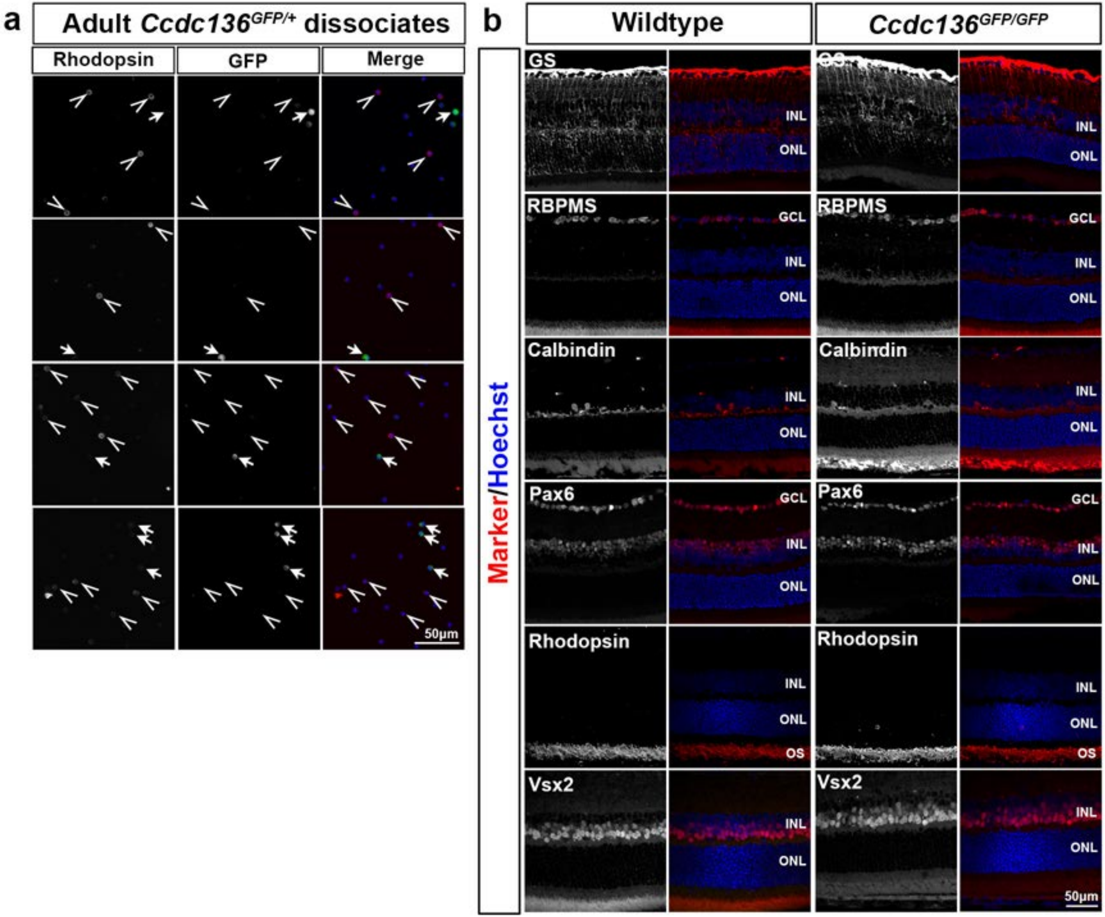

a
Adult Ccdc136GFP/+ dissociates
Rhodopsin
GFP
Merge
50µm
b
Wildtype
Ccdc136GFP/GFP
Marker/Hoechst
GS
INL
ONL
RBPMS
GCL
INL
ONL
Calbindin
INL
ONL
Pax6
GCL
INL
ONL
Rhodopsin
INL
ONL
OS
Vsx2
INL
ONL
50µm

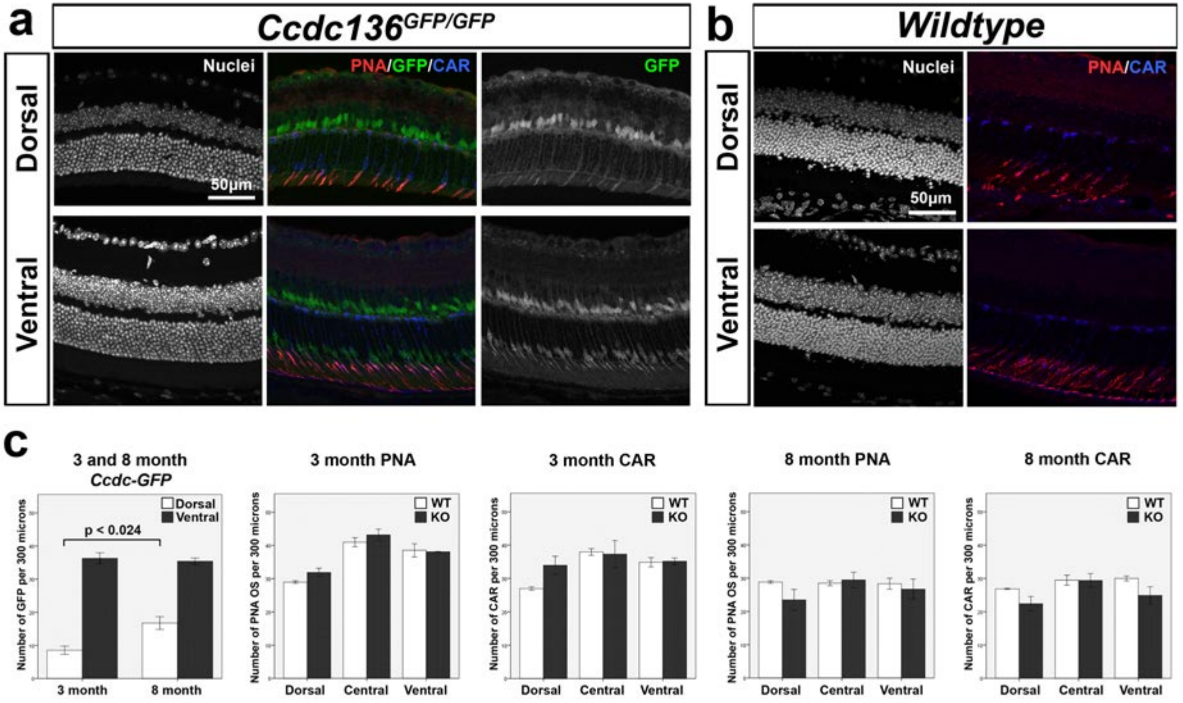
a
Ccdc136GFP/GFP
b
Wildtype
Dorsal
Ventral
Nuclei
PNA/GFP/CAR
GFP
PNA/CAR
50µm
c
3 and 8 month
Ccdc-GFP
3 month PNA
3 month CAR
8 month PNA
8 month CAR
Dorsal
Ventral
WT
KO
p < 0.024
Number of GFP per 300 microns
Number of PNA OS per 300 microns
Number of CAR per 300 microns
3 month
8 month
Dorsal
Central
Ventral

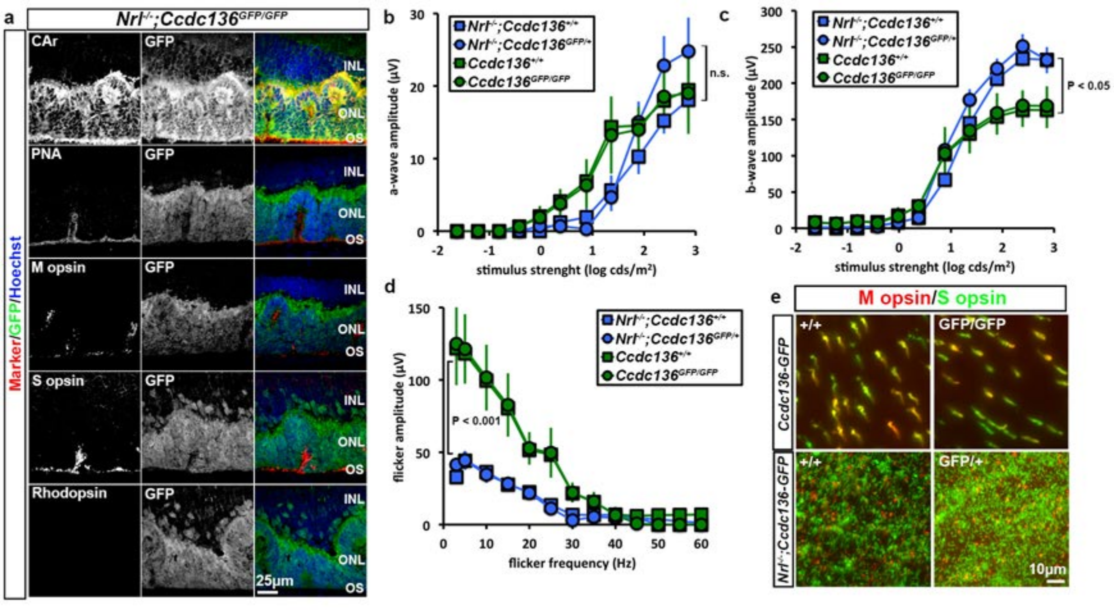

a
Nrl-/-;Ccdc136GFP/GFP
Marker/GFP/Hoechst
CAr
PNA
M opsin
S opsin
Rhodopsin
GFP
INL
ONL
OS
25μm
b
a-wave amplitude (μV)
stimulus strenght (log cds/m²)
Nrl-/-;Ccdc136+/+
Nrl-/-;Ccdc136GFP/+
Ccdc136+/+
Ccdc136GFP/GFP
n.s.
c
b-wave amplitude (μV)
P < 0.05
d
flicker amplitude (μV)
flicker frequency (Hz)
P < 0.001
e
M opsin/S opsin
Ccdc136-GFP
Nrl-/-;Ccdc136-GFP
+/+
GFP/GFP
GFP/+
10μm

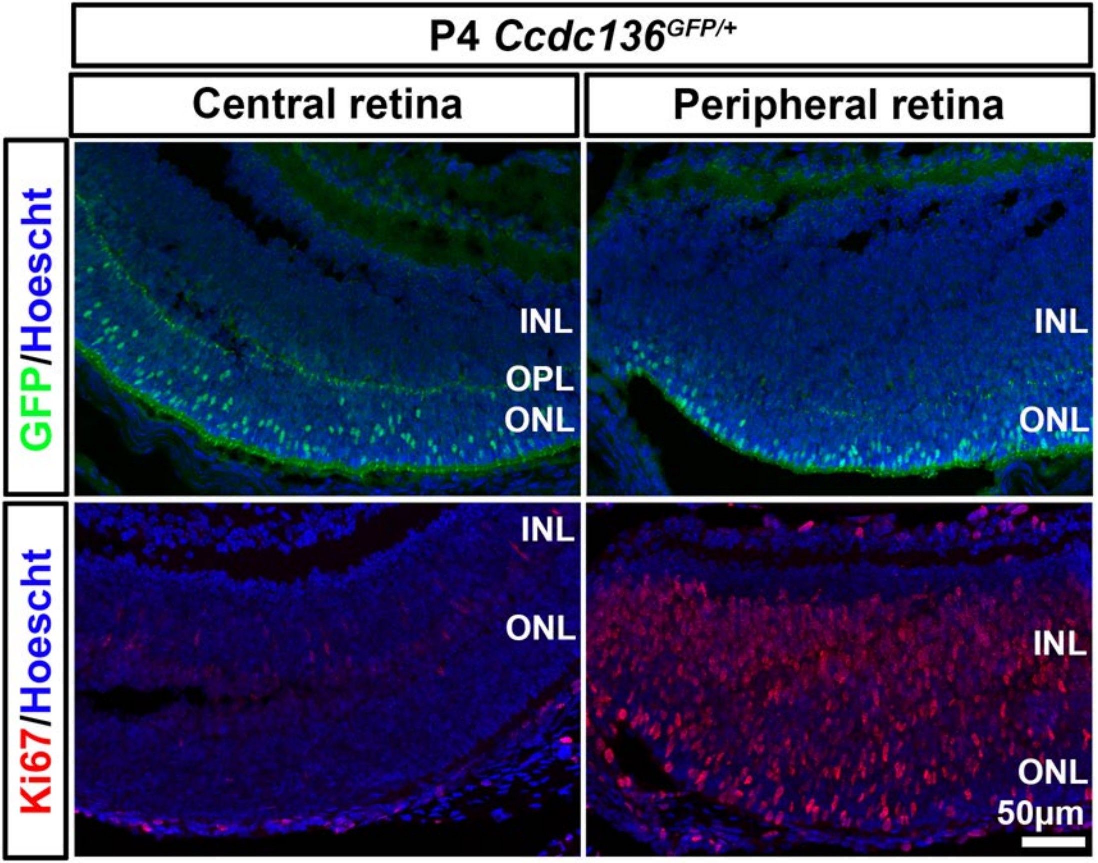
P4 Ccdc136GFP/+
Central retina
Peripheral retina
GFP/Hoescht
Ki67/Hoescht
INL
OPL
ONL
INL
ONL
INL
ONL
INL
ONL
50μm

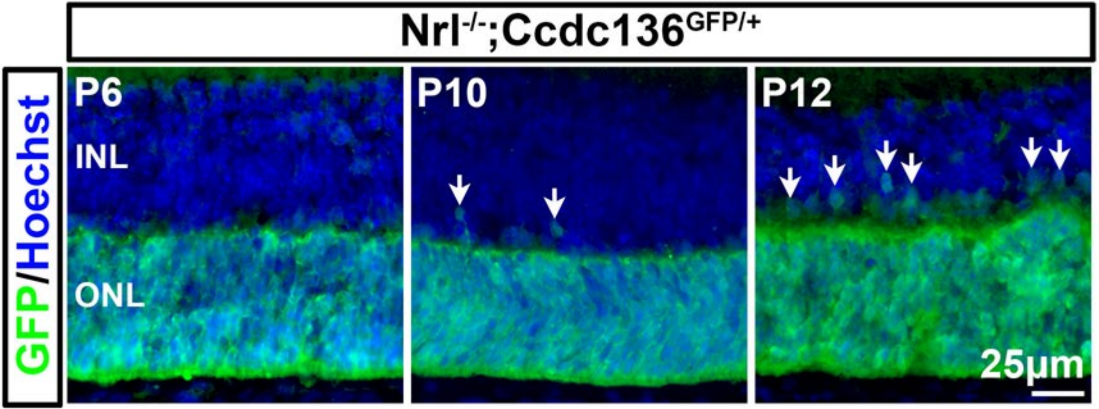

Nrl-/-;Ccdc136GFP/+
GFP/Hoechst
P6
INL
ONL
P10
P12
25μm

a

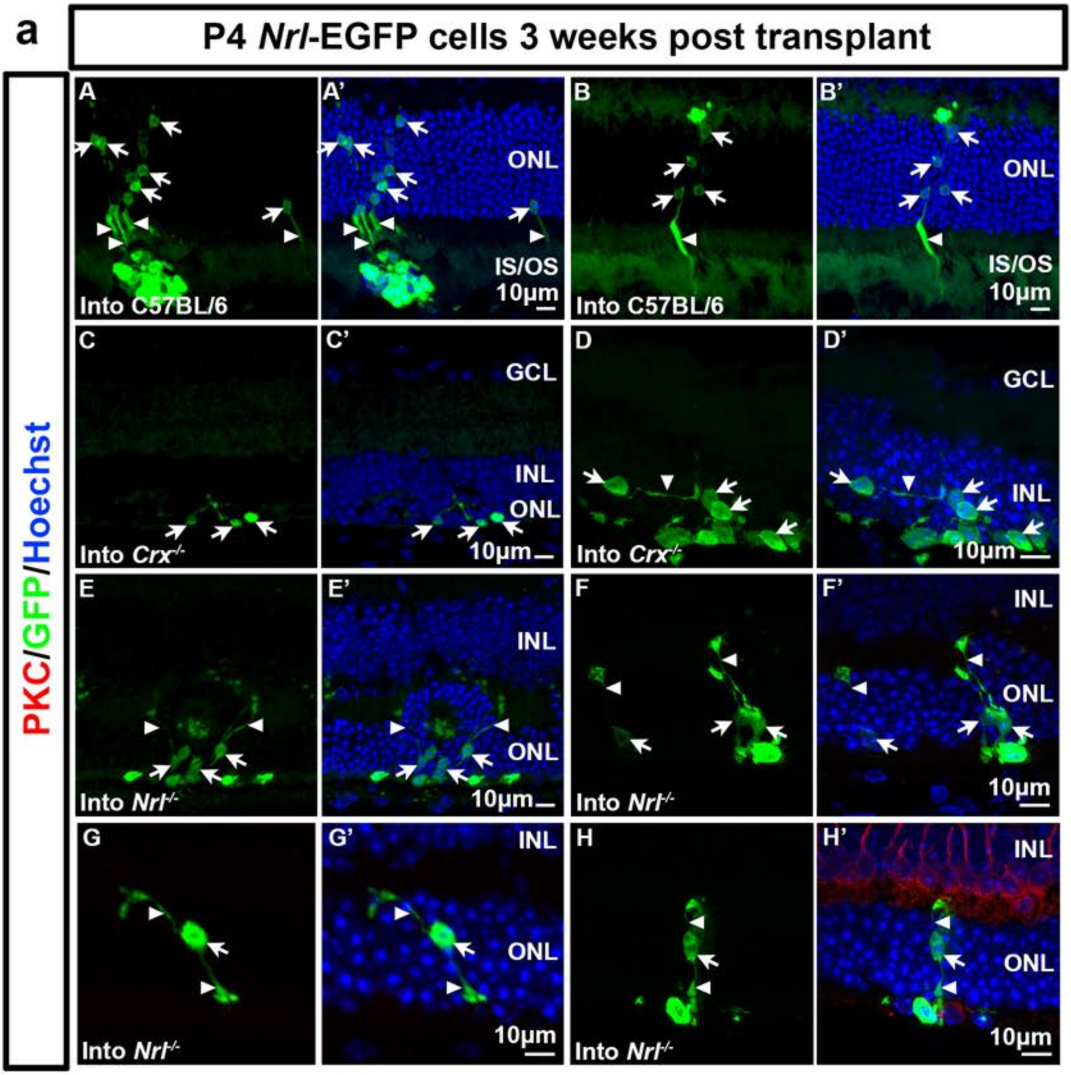

P4 Nrl-EGFP cells 3 weeks post transplant
PKC/GFP/Hoechst
A
A'
B
B'
ONL
IS/OS
10μm
Into C57BL/6
C
C'
D
D'
GCL
INL
ONL
Into Crx-/-
E
E'
F
F'
Into Nrl-/-
G
G'
H
H'

b

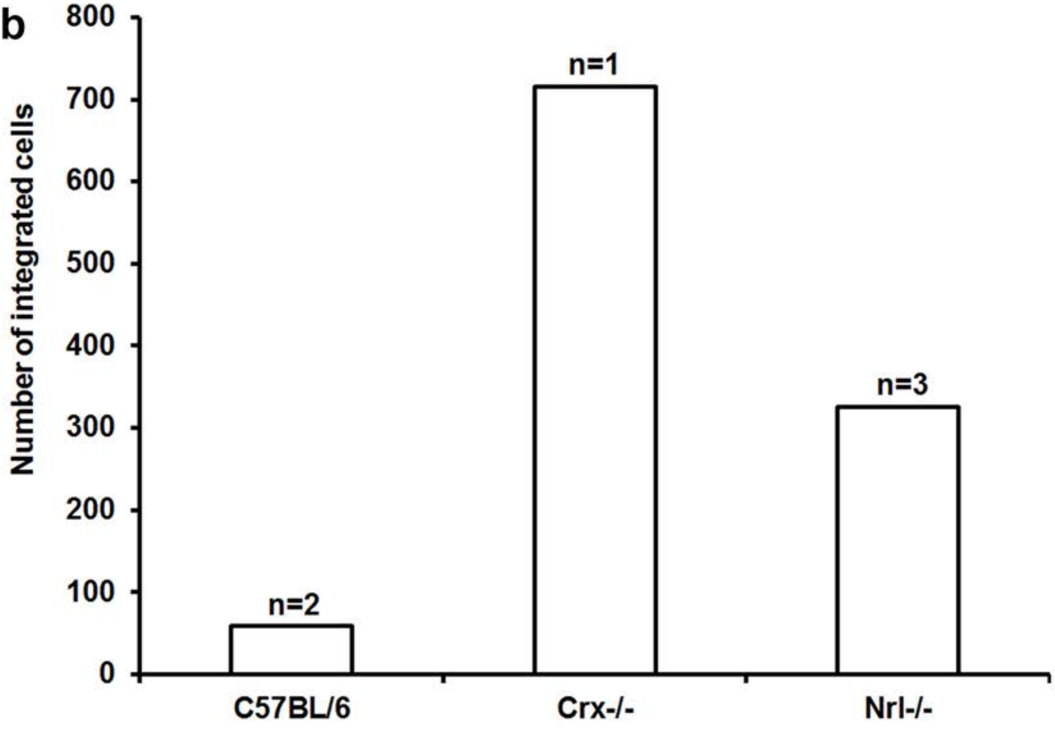

Number of integrated cells
800
700
600
500
400
300
200
100
0
n=2
n=1
n=3
C57BL/6
Crx-/-
Nrl-/-

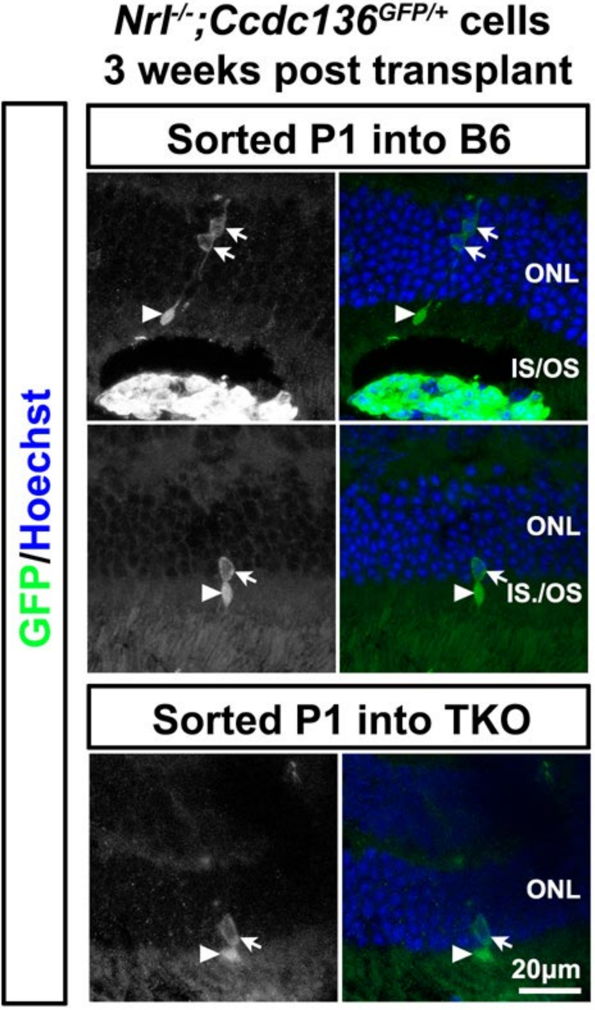
Nrl-/-;Ccdc136GFP/+ cells
3 weeks post transplant
Sorted P1 into B6
ONL
IS/OS
ONL
IS./OS
Sorted P1 into TKO
ONL
20μm
GFP/Hoechst

P1 *Nrl$^{-/-}$;Ccdc136$^{GFP/-}$* cells 3 weeks post transplant

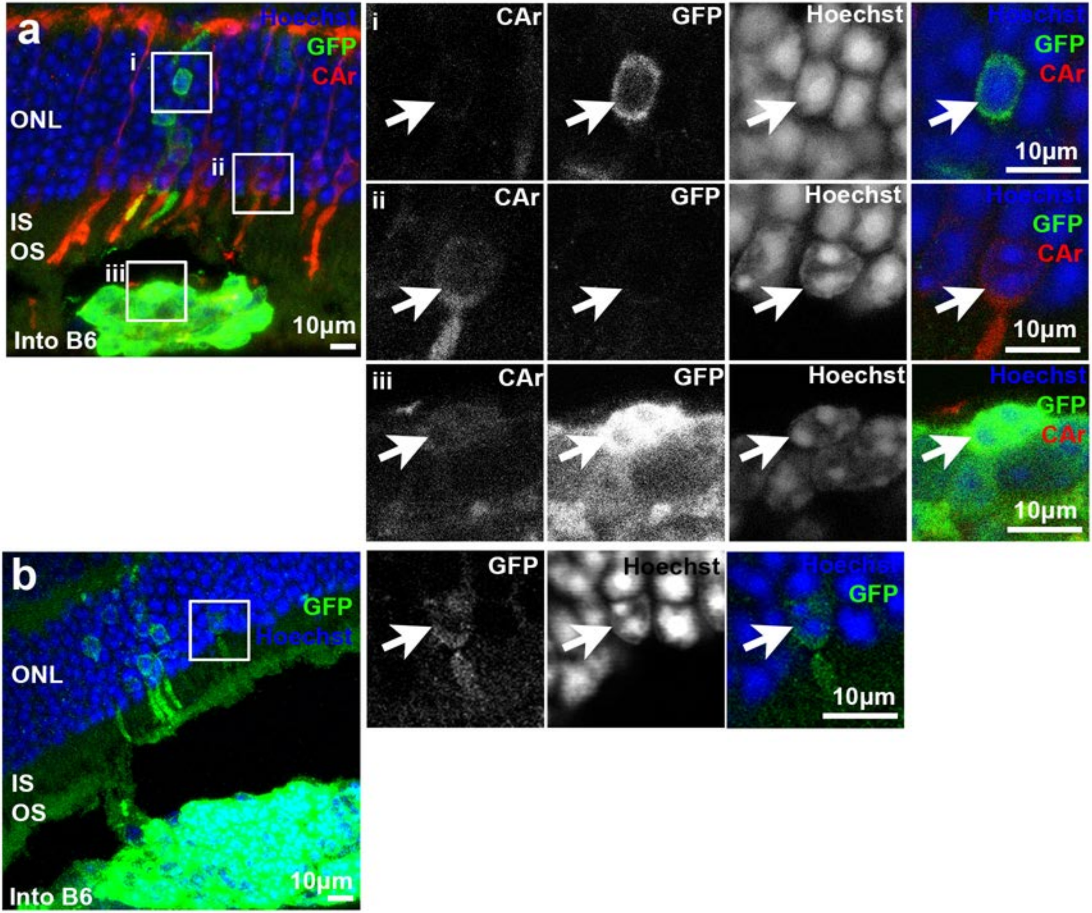

Supplement: Supplementary Information [file srep22867-s1.pdf]
